# Temporal Dynamics of the Interaction between Reward and Time Delay during Intertemporal Choice

**DOI:** 10.3389/fpsyg.2016.01526

**Published:** 2016-10-12

**Authors:** Dan-Yang Gui, Jin-Zhen Li, Xiaoli Li, Yue-jia Luo

**Affiliations:** ^1^Institute of Affective and Social Neuroscience, Shenzhen University, ShenzhenChina; ^2^Institute of Psychology, Chinese Academy of Sciences, BeijingChina; ^3^State Key Laboratory of Cognitive Neuroscience and Learning & IDG/McGovern Institute for Brain Research, Beijing Normal University, BeijingChina; ^4^Department of Psychology, Ningbo University, NingboChina

**Keywords:** ERP, temporal discounting, subjective value, economic decision-making, delayed discounting task

## Abstract

Intertemporal choice involves the processes of valuation and choice. Choice is often the result of subjective valuation, in which reward is integrated with time delay. Here, using event-related potential (ERP) signals as temporal hallmarks, we aim to investigate temporal dynamics of how reward interacts with time delay during a delayed discounting task. We found that participants preferred immediate rewards when delayed rewards were small or over long-term delays. Our ERP results suggested that the P200 component reflected an initial valuation of reward and time delay, while the frontal N2 component correlated with individual choices of immediate option of rewards. The LPP component was modulated by the N2 component. These findings demonstrate that the N2 component is the key component in temporal dynamics of the interaction between reward and time valuation.

## Introduction

Temporal discounting is a phenomenon in which subjective valuation of a reward declines with delay, until the delivery of the reward is increased (Samuelson, 1937; Green and Myerson, 2004). The delay discounting task, which involves a choice between a smaller, more immediate reward and a larger, more delayed reward, has been used widely to investigate the underlying mechanism of temporal discounting. A substantial amount of research has shown that individuals prefer to choose an immediate reward rather than a delayed one, and that the value of the soonest available reward is subjectively overvalued (Berns et al., 2007; Peters and Büchel, 2011). Temporal discounting plays an important role in risk and impulsive decision-making, and individuals vary widely in the rate at which they discount future rewards. These variations correlate with real-life behaviors and clinical disorders involving self-control, such as drug abuse, gambling, and addition, and also attention-deficit/hyperactivity disorder and Parkinson’s disease (Reynolds, 2006; Scheres et al., 2006; Milenkova et al., 2011; Yi and Landes, 2012).

A specific network of brain regions recruited for temporal discounting has been identified in previous neuroimaging studies. The neural mechanisms underlying temporal discounting may involve two processing stages: valuation and choice (Peters and Büchel, 2011; Liu et al., 2012). The valuation process involves the neural computation and representation of the subjective values of the available decision options, and activates brain regions in ventral striatum (VS), orbitofrontal cortex (OFC), ventromedial prefrontal cortex (vmPFC), and posterior cingulate cortex (PCC) (McClure et al., 2004; Kable and Glimcher, 2007, 2010; Ballard and Knutson, 2009; Padoa-Schioppa and Cai, 2011; Liu and Feng, 2012; Liu et al., 2012). The choice process comprises sub-processes that include conflict monitoring, cognitive control, and prospection that lead to and support the selection of the action. Brain regions activated by choice include the dorsolateral prefrontal cortex (DLPFC), anterior cingulate cortex (ACC), lateral parietal cortex, and hippocampus (McClure et al., 2004; Kable and Glimcher, 2007; Hare et al., 2009; Peters and Büchel, 2009, 2010; Cai et al., 2011).

Although spatial neural networks have been studied extensively using functional magnetic resonance imaging (fMRI) and lesion studies, little is known of the temporal dynamics of neural activity of intertemporal choice. Previous event-related brain potential (ERP) studies on decision-making task showed that the frontal P200 component might reflect stimulus evaluation and quick assessment (Potts et al., 2006). The peak amplitudes of P200 and P300 varied, as the temporal distance for the reward increased from 2 weeks to 50 years (He et al., 2012). Harris et al. (2013) found evidence for top-down attention filtering early on the decision period (150–200 ms poststimulus onset), and value modulation later in the process (450–650 ms poststimulus onset) (Harris et al., 2013). In our previous study (Li et al., 2012), we found that the ERP components P200 and N2 might be the key factors that determine the discounting behaviors of survivors in Wenchuan earthquake during delay discounting task.

One important issue is when objective valuation is modulated to subjective valuation (integrated valuation) in delay discounting decision-making, individuals should have an initial detection and evaluation about objective values of rewards (e.g., absolute money magnitudes or action consequences) (Nikolaev et al., 2008; Boudreau et al., 2009; Chen et al., 2009). And, then objective valuation might be shaped to subjective valuation when time delays are integrated into the objective value of money rewards. However, when and how reward and time delay interacted remains unknown.

In the present study, we developed a modified, parametrically orthogonalized delay discounting task (money rewards vs. time delays) that was adapted specifically for an event-related brain potential study. Thus, this design enabled us to investigate the electrophysiological correlates of how the reward valuation processes interact with the delay valuation processes. Given the low temporal resolution of fMRI, the temporal dynamics of neural activity of intertemporal choice can be systematically investigated by ERP, which overcomes the temporal drawbacks of fMRI studies. According to previous studies, the frontal P200 component might reflect stimulus evaluation and a quick assessment (Potts et al., 2006; Boudreau et al., 2009; Chen et al., 2009). We predicted that small and large rewards would have different amplitudes of the P200 component. Self-control in decision-making could modulate valuation system (Hare et al., 2009), frontal N2 component linked to the cognitive control process (Folstein and Van Petten, 2008), we predicted that objection valuation would be shaped to subjective valuation when time delays integrate into the objective value of money rewards in N2, P300 or later component of processing.

## Materials and Methods

### Participants

A total of 34 healthy volunteers from Beijing Normal University were recruited for this study, four subjects were excluded due to recording errors and severe artifacts in the EEG data. Therefore, brain activity from 30 participants (18 females; mean age 21.13 ± 1.88 years) was fully analyzed. All participants were right-handed, had normal vision (with or without correction), reported no history of affective disorders or neurological diseases, and did not regularly use medications. All participants gave written informed consent before the experiment, the study protocol was approved by local ethics committee of Beijing Normal University. All methods were carried out in accordance with the approved protocol.

### Procedures

**FIGURE 1:**
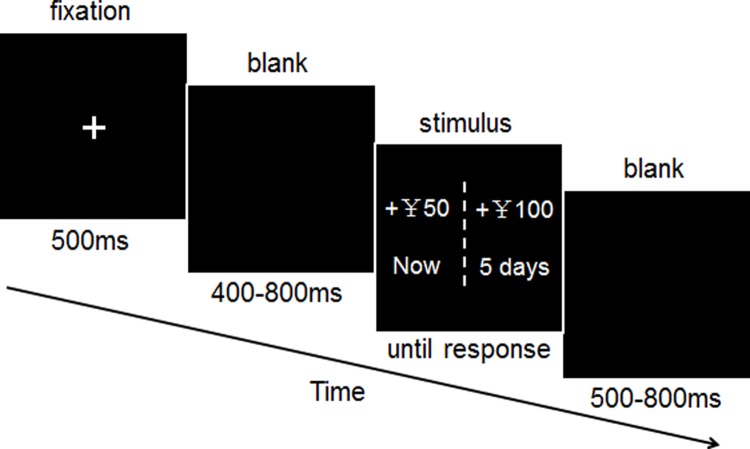
**Time course of a single delay discounting trial.** Each trial began with a 500-ms fixation point and was followed by the blank screen, randomized between 400 and 800 ms. A screen displaying the stimulus presentation was then shown until the participants responded. The inter-trial interval was randomized to between 500 and 800 ms.

The procedure for the task is shown in **Figure 1**. The participants performed the experimental tasks in a small, sound-attenuated, and electrically shielded room. The display of the stimuli and acquisition of behavioral data were conducted by E-Prime software (Version 1.1, Psychology Software Tools, Inc.) and were presented on a CRT monitor, with an 80-Hz refresh rate. During the task, the participants were positioned approximately 80 cm from the computer screen.

The entire experiment comprised of 240 test trials and eight practice trials. Participants were instructed to choose between two monetary-gain alternatives; an immediate and smaller reward (IS) or a later and larger reward (LL), to be obtained at different times (e.g., now vs. 5 days later). For each set of intertemporal alternatives, the IS-money reward was fixed to ¥ 50, and the delayed-money reward was randomly picked from a predetermined series of monetary amounts: small reward (a 20% increase compared with the IS): ¥ 60; large reward (a 100% increase compared with the IS): ¥ 100. The later time points were randomly picked from a predetermined series of delayed periods [short term delays (S-TD): 1 day later, 3 days later, 5 days later; long term delays (L-TD): 6 months later, 9 months later, 1 year later]. The two alternatives for each choice were presented on either side of the screen. The location of the immediate and delayed options were randomly assigned (left or right) on each trial and were counterbalanced across trials, and participants were instructed to press the “F” key to denote a left-side choice or the “J” key to denote a right-side choice. Participants were informed prior to the task that they would receive actual payments based on their choices. One of the choices the participants made was selected at random to determine his or her payoffs. If the randomly selected choice was an immediate reward, the participant was paid in cash at the end of the experiment. If the randomly selected option was a delayed reward, the participant would receive the monetary reward at a later date determined by the amount of delay specified in the option. Additionally, all methods were carried out in accordance with the approved protocol.

### EEG Recording and Analysis

Electroencephalograms (EEG) were recorded from 64 scalp sites, using electrodes mounted on an elastic cap (NeuroScan Inc.), with an online reference to the left mastoid. The horizontal electrooculogram (HEOG) was recorded from two electrodes placed laterally to the right and left eyes. The vertical electrooculogram (VEOG) were recorded from electrodes placed above and below the right eye. All inter-electrode impedances were maintained below 5 kΩ. All signals were sampled at 500 Hz and band-pass filtered within a 0.01–100 Hz frequency range.

During off-line analyses, all EEG signals were re-referenced to the mean of the left and right mastoids. The EEG data were low-pass filtered below 20 Hz (24 dB/oct). Ocular artifacts were removed from the data using a regression procedure implemented with Neuroscan software (Semlitsch et al., 1986). Trials containing EEG sweeps with amplitudes exceeding ±80 μV were excluded. For each stimulus, epochs of 1000 ms in duration, including a 200 ms pre-stimulus period used as baseline, were extracted from the continuous EEG record.

Repeated measures analyses of variance (ANOVAs) for all ERP components were conducted for magnitude (small reward vs. large reward) by time delay (short-term vs. long-term delay) as within-participants factors. An additional within-participants factor was the electrode. Frontal P200 component was measured as a peak amplitude within the post-stimulus time windows of 150 to 250 ms on electrodes Fz, F3, F4, FCz, FC3, and FC4. Frontal N2 component was measured as a peak amplitude within the post-stimulus time windows of 250 to 350 ms on electrodes Fz, F3, F4, FCz, FC3, and FC4. P300 component was measured as the mean amplitude during 400 to 500 ms on electrodes Pz, P3, P4, CPz, CP3, and CP4. Left and right orbitofrontal Late Positive Potential (LPP; mean amplitude during 500 to 700 ms) and orbitofrontal P200 (peak amplitude during 150–250 ms) components were measured on electrodes AF7, AF3, FP1 for the left and AF8, AF4, FP2 for the right. These electrode sites were chosen based on previous literature and visual inspection of the ERP grand average waveforms for all conditions (Kok, 2001; Polich, 2007; Folstein and Van Petten, 2008).

For all statistical analyses using SPSS version 13.0 (SPSS, Inc., Chicago, IL, USA), the significance level was set to 0.05. Greenhouse–Geisser correction for non-sphericity was applied as appropriate. *Post hoc* tests for multiple comparisons were corrected by Bonferroni method. Significant interactions were analyzed by simple-effects models. Partial eta-squared was reported to demonstrate the effect size of the statistical results. Pearson correlations were performed between magnitudes of ERP components. Because the percentage of immediate choices was not normally distributed, we performed Spearman’s correlations (non-parametric test) between magnitude of ERP components and behavioral responses. The interrelationship among those variables helped elaborate the functional significance of the ERP components obtained in current study.

## Results

### Choices of Immediate Option of Rewards

**FIGURE 2:**
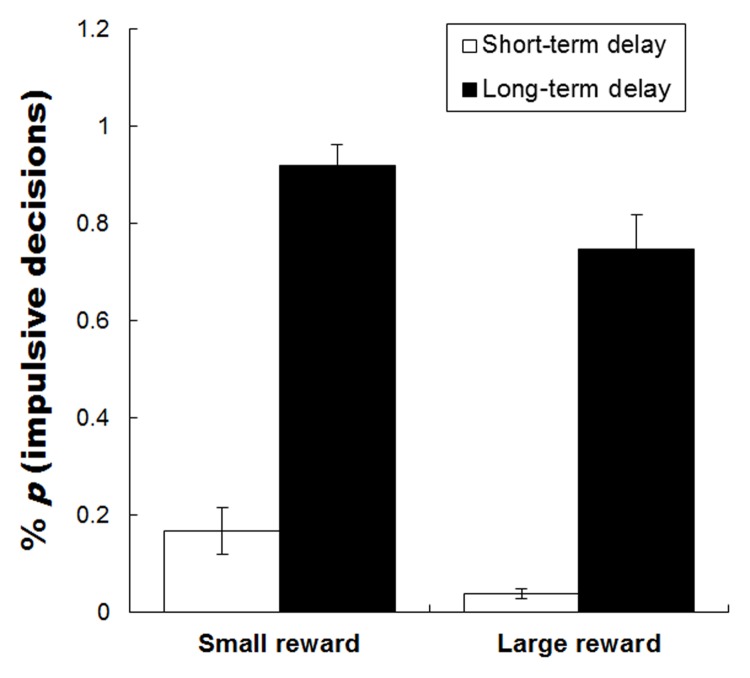
**The mean percentage choice for an immediate option of reward during the four task conditions.** Error bars denote standard error of the mean.

The percentages of immediate choices during the four task conditions are shown in **Figure 2**. Repeated measures analyses of variance (ANOVAs) on choices of immediate option of rewards were conducted using the magnitude (small reward vs. large reward) by time delay (short-term vs. long-term delay) as within-participants factors. The results revealed a significant main effect of money magnitude on the percentage of immediate choices [*F*(1,29) = 15.827, *p* < 0.001, $\eta_{p}^{2}$ = 0.353], immediate choices for small rewards were chosen more often than large rewards. This indicates that participants preferred immediate rewards (involving impulsive decisions) when delayed rewards were small. The main effect of time delay on the percentage of immediate choices was also significant [*F*(1,29) = 185.681, *p* < 0.001, $\eta_{p}^{2}$ = 0.865], immediate choices for long-term time delays were chosen more often than short-term time delays. This implies that participants preferred immediate rewards (involving impulsive decisions) over long-term delays, and that the subjective valence of the money was discounted more severely when money was delayed over the long-term. There was no significant interaction between magnitude and time delay.

Mean response times (RTs) ± standard errors of the choices were 1441 ± 151 ms (small reward S-TD), 1304 ± 137 ms (small reward L-TD), 1268 ± 152 ms (large reward S-TD), 1271 ± 109 ms (Large reward L-TD). ANOVAs on RTs of the choices revealed that there was no significant main effects of magnitude [*F*(1,29) = 0.663, *p* = 0.422, $\eta_{p}^{2}$ = 0.023]/time delay [*F*(1,29) = 0.563, *p* = 0.459, $\eta_{p}^{2}$ = 0.020], and also no significant interaction between magnitude and time delay [*F*(1,29) = 0.878, *p* = 0.357, $\eta_{p}^{2}$ = 0.030].

### ERP Results

#### P200

**FIGURE 3:**
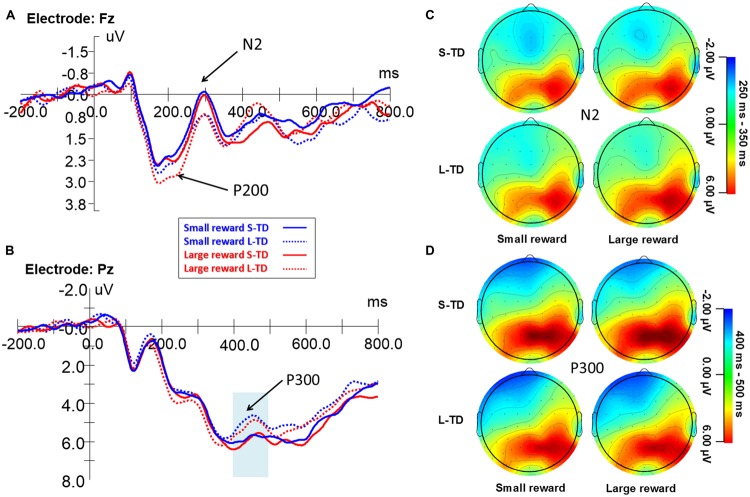
**Grand-averaged ERP waveforms at the electrode Fz **(A)** and Pz **(B)** during the four task conditions.** Topographic maps for the N2 **(C)** and P300 **(D)**. ERPs signals were time-locked to the stimulus presentation onset – time point = 0 on the *x*-axis of figures **(A,B)**.

**Figure 3** shows the ERP waveform for the frontal P200 during the four task conditions. A significant main effect of money magnitude was observed on the P200 amplitude [*F*(1,29) = 4.915, *p* = 0.035, $\eta_{p}^{2}$ = 0.145], in which amplitudes for large-reward conditions were significantly greater than those for small-reward conditions. A main effect of time delay on the P200 amplitude was also significant [*F*(1,29) = 5.848, *p* = 0.022, $\eta_{p}^{2}$ = 0.168], in which amplitudes for long-term delays (involving more impulsive decisions) were significantly greater than those for short-term delays, and there was no significant interaction between magnitude and time delay.

**FIGURE 4:**
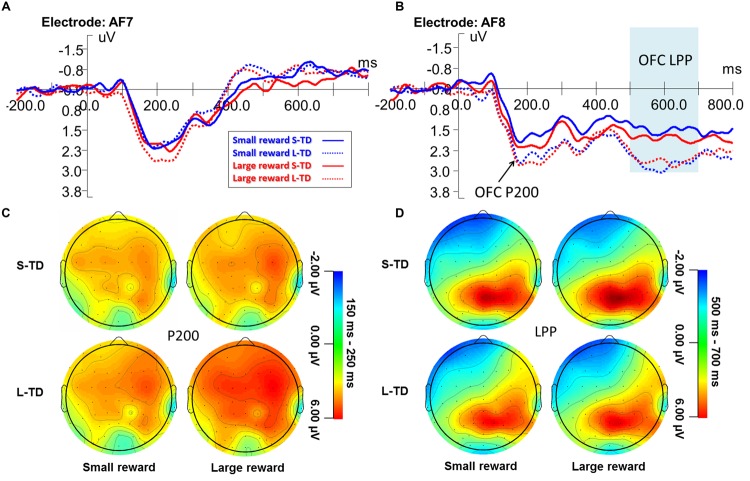
**Grand-averaged ERP waveforms at the orbitofrontal electrode AF7 **(A)** and AF8 **(B)** during the four task conditions.** Topographic maps for the P200 **(C)** and LPP **(D)**. ERPs signals were time-locked to the stimulus presentation onset – time point = 0 on the *x*-axis of figures **(A,B)**.

**Figure 4** shows the ERP waveform for the orbitofrontal P200 during the four task conditions. ANOVAs revealed a significant main effect of money magnitude on the right P200 amplitude [*F*(1,29) = 4.664, *p* = 0.039, $\eta_{p}^{2}$ = 0.139], amplitudes during large-reward conditions were significantly greater than those during small-reward conditions. A main effect of time delay on the right P200 amplitude was significant [*F*(1,29) = 12.662, *p* = 0.001, $\eta_{p}^{2}$ = 0.304], and amplitudes of long-term delays (involving more impulsive decisions) were significantly greater than those of short-term delays with no significant interaction between magnitude and time delay observed. There were no significant main effects of either magnitude or time or interaction observed for the left orbitofrontal P200 component. There was no significant correlation between the percentages of immediate choices and P200 peak amplitudes. This indicated that the objective values of money and time were distinguished, respectively.

#### N2

**Figure 3** shows the ERP waveform for the N2 component during the four task conditions. ANOVAs revealed a significant main effect of time delay on the N2 amplitude [*F*(1,29) = 6.859, *p* = 0.014, $\eta_{p}^{2}$ = 0.191], amplitudes for the short-term delays (involving more non-impulsive decisions) were significantly more negative than those for long-term delays (involving more impulsive decisions). There was no significant main effect of money magnitude on the N2 amplitude [*F*(1,29) = 1.648, *p* = 0.209, $\eta_{p}^{2}$ = 0.054] and no significant interaction between magnitude and time delay. This indicated that the value of money was integrated with the value of time.

Significant correlations were obtained between the percentages of immediate choices and mean N2 peak amplitudes of all conditions on FCz (*r* = -0.415, *p* = 0.011) and nearly significant correlation on mean of all selected electrodes (*r* = -0.205, *p* = 0.056), indicating that the N2 amplitudes over the frontal region may predict individual’s choice. Specifically, significant correlations were obtained between the percentages of immediate choices and N2 peak amplitudes on Fz (*r* = -0.398, *p* = 0.015), F3 (*r* = -0.322, *p* = 0.041), F4 (*r* = -0.467, *p* = 0.005), FCz (*r* = -0.521, *p* = 0.002), FC3 (*r* = -0.36, *p* = 0.025) and FC4 (*r* = -0.545, *p* = 0.001) and mean of N2 peak amplitudes of all selected electrodes (*r* = -0.456, *p* = 0.006) for large reward L-TD; Moreover, negative correlations between the percentages of immediate choices and N2 peak amplitudes on FCz electrode for small reward S-TD time delays was significant (*r* = -0.223, *p* = 0.044). The insignificance of correlation between behavioral results and N2 peak amplitudes for the other two task conditions might be due to limited variance of participants’ behavioral responses for the two conditions (mean = 0.91, *SD* = 0.24; mean = 0.03, *SD* = 0.05). There were no other significant correlations between the percentages of immediate choices and other ERP components.

#### P300

**Figure 3** shows the ERP waveform for the P300 component during the four task conditions. ANOVAs revealed a significant main effect of time delay on the P300 amplitude [*F*(1,29) = 5.944, *p* = 0.021, $\eta_{p}^{2}$ = 0.170], amplitudes for short-term delays (involving more non-impulsive decisions) were significantly greater than those for long-term delays (involving more impulsive decisions). There was no significant main effect of money magnitude on the P300 amplitude [*F*(1,29) = 0.522, *p* = 0.476, $\eta_{p}^{2}$ = 0.018] and no interaction between magnitude and time delay. There was no significant correlation between the percentages of immediate choices and P300 amplitudes.

#### LPP

**Figure 4** shows the ERP waveform for the left and right LPP during the four task conditions. ANOVAs revealed a significant main effect of time delay on the right LPP amplitude [*F*(1,29) = 11.806, *p* = 0.002, $\eta_{p}^{2}$ = 0.289], amplitudes for short-term delays (involving more non-impulsive decisions) were significantly greater than those for long-term delays (involving more impulsive decisions), but there was no significant main effect of time delay on the left-LPP amplitude [*F*(1,29) = 0.004, *p* = 0.948, $\eta_{p}^{2}$ < 0.001]. Main effects of magnitude on both the left- and right-LPP amplitude were not significant, and no other significant interaction between magnitude and time delay was found on right- and left-LPP amplitude. There was no significant correlation between the percentages of immediate choices and LPP amplitudes. These results indicated that the LPP component of decision-making during the delay discounting task showed right hemisphere laterality.

**FIGURE 5:**
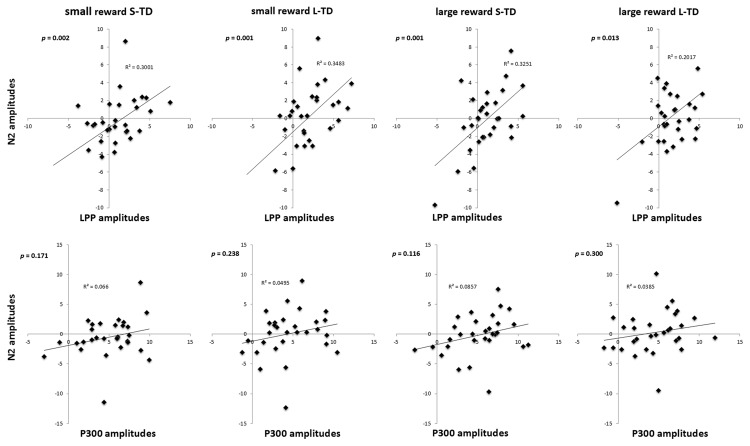
**Correlations between the N2 and the LPP, P300.** The right-LPP amplitudes were significantly correlated with the amplitudes of N2 in small reward S-TD, small reward L-TD, large reward S-TD, and large reward L-TD conditions. There was no significant correlation between amplitudes of N2 and P300.

The right-LPP amplitudes were significantly correlated with the amplitudes of N2 component in four conditions of small reward S-TD (*r* = 0.548, *p* = 0.002), small reward L-TD (*r* = 0.590, *p* = 0.001), large reward S-TD (*r* = 0.570, *p* = 0.001) and large reward L-TD (*r* = 0.449, *p* = 0.013). There was no significant correlation between amplitudes of N2 and P300 (**Figure 5**). These results indicated that the right-LPP amplitude was modulated by N2 amplitude.

## Discussion

In the present study, we investigated the electrophysiological correlates of when and how reward interacted with time delay, using a delay discounting paradigm. We found that the main effects of time delay and reward appeared at a different time course during an intertemporal choice. Our findings showed that the P200 component was associated with initial stimulus identification and evaluation (objective valuation), while the frontal N2 component was correlated individual difference of choice that associated with subjective valuation. Further, the right orbitofrontal LPP component might be related to further valuation that modulated by N2 component.

Behavioral results indicate that participants preferred immediate rewards (involving impulsive decisions) when rewards were small, or over long-term delays. The results are consistent with previous studies (Frederick et al., 2002; Berns et al., 2007). This suggests that subjective values of small rewards are discounted more quickly than large rewards, and subjective value of the money is discounted more severely when delayed over the long-term.

The frontal and orbitofrontal P200 component showed a significant main effect of both time delay and reward but no other interaction between them (**Figures 3** and **4**). This result indicates that the subjects detected the initial objective value of money magnitude and time, respectively, but did not integrate these factors. Previous ERP studies on cognition and decision-making showed that the frontal P200 component might reflect stimulus evaluation and a quick assessment (Potts et al., 2006; Nikolaev et al., 2008; Boudreau et al., 2009; Chen et al., 2009). The probable sources of the frontal P200 component might be the mesotelencephalic dopamine reward system and medial frontal cortex for the identification of task-relevant perceptual representations (Potts et al., 2006). Consistent with those studies, our results suggest that in the early stage valuations of both time delays and money rewards are specifically distinguished. Our findings are also consistent with single valuation account, in which Kable argued that a single neural system (i.e., medial PFC, VS, or PCC) represents the value of all rewards regardless of delay (Kable and Glimcher, 2007, 2010).

After the initial objective valuation was processed in P200 stage, the significant main effect of money magnitude disappeared in the N2 component. And N2 component was specifically correlated with individuals’ differences of choices. Our findings indicate that N2 component is the key component in time course of interaction between time delay and reward valuation. In this stage, the objective valuation of money magnitude is then integrated with time delay, and that the objective valuation of time delay is also integrated with money rewards. This integration of reward and time may give rise to a new subjective valuation, which could be modulated by other cognitive processes, such as self-control and projection (Hare et al., 2009; MacKillop et al., 2011; Peters, 2011). N2 peak amplitudes in brain electrophysiological activities may be one of key biomarkers of impulsivity and predictors of individual’s intertemporal choices.

The P300 component is one of the most frequently studied components of ERPs. The P300 and P300-related late activity in ERP studies are typically regarded as a measure to investigate various cognitive processes, processing capacity and mental workload (Kok, 2001; Polich, 2007). Advanced cognitive processes (i.e., evaluation and stimulus categorization, memory encoding and updating, making decisions under complex social context) are embodied in the P300 or late positive component (Chen et al., 2009; Paynter et al., 2009; Beste et al., 2012; Mathes et al., 2012). In this study, our results found that the P300 amplitudes of S-TD (involving more non-impulsive decisions) were significantly larger than those of L-TD (involving more impulsive decisions), suggesting that more attentional and controlled cognitive processing resources are required for cost-benefit evaluation and computation that eventually favor non-impulsive decisions. Moreover, larger P300 amplitudes for non-impulsive decisions may involve more future projection memory processing. These results are consistent with fMRI studies that reported decisions for delayed rewards involve more controlled cognitive processes (greater relative fronto-parietal activity; McClure et al., 2004) and future-minded memory thinking (Peters and Büchel, 2010).

The orbitofrontal LPP component is modulated by N2 component, one possible source of this component is OFC. An overwhelming amount of evidence has shown that the OFC plays a fundamental role in value-based decision-making (Tremblay and Schultz, 1999; Padoa-Schioppa and Assad, 2006; Schoenbaum et al., 2006; Burke et al., 2008; Jones et al., 2012). Neurons of the OFC in many species, including rats, monkeys and humans, encode the preferred value of outcomes and actions (Cai et al., 2011; Peters and Büchel, 2011). Concordantly, many cross-species lesion studies have revealed that damage to the OFC impairs various aspects of valued-based decision-making (Padoa-Schioppa and Cai, 2011; Wallis, 2012). Harris et al. (2013) found evidence for value modulation later in the process (450–650 ms poststimulus onset). This study also revealed significant causal connectivity from DLPFC to vmPFC from 500 to 650 ms, suggesting that later valuation was modulated by DLPFC (Harris et al., 2013). Our results are consistent with previous studies which showed that later valuation in the process may be modulated by early component.

Although we found a robust difference in effects of the ERP components between the left and right orbitofrontal region, both the orbitofrontal P200 and LPP showed an obvious right orbitofrontal laterality. We do not think the right laterality is due to experimental design, because the location of immediate and delayed options were randomly assigned (left or right) on each trial, and participants’ responses were also counterbalanced. The specific functional role of the right OFC in intertemporal choice or value-based decision-making remains unclear. The findings on the right OFC have been equivocal. Zatorre et al. (1992) reported that the human brain favored the right OFC area for olfactory processing (Zatorre et al., 1992). Since then, others have demonstrated that the right posterior medial OFC involved particularly in the processing of negative outcomes of action (Szatkowska et al., 2011), such as decisions involving eating behavior. Woolley et al. (2007) reported that binge eating could occur despite reported satiety and might be associated with damage to the right side of the orbitofrontal-insular-striatal circuit in humans (Woolley et al., 2007). Whereas Suda et al. (2010) reported that eating behavior problems could be correlated with the left OFC (Suda et al., 2010). There have been very few studies of lesions to the right OFC on value-based decision-making. Further studies should focus on the functional specificity of the right OFC on value-based decision-making, and how this may be altered when this region is lesioned.

To sum up, in the current study, we used a delayed discounting task to investigate temporal dynamics of how reward interacts with time delay. The behavioral results found that that participants preferred immediate rewards (involving impulsive decisions) when delayed rewards were small or over long-term delays. The ERP results manifested that frontal and orbitofrontal P200 components reflected an initial valuation of the stimulus and a quick assessment that might involve the mesotelencephalic dopamine reward system and the medial frontal cortex. The frontal N2 component correlated with individual choices of immediate option of rewards. The LPP component was modulated by the N2 component. These findings extend knowledge about the temporal processing of intertemporal decision-making, and indicate that the N2 component is the key component in temporal dynamics of the interaction between reward and time valuation.

## Author Contributions

Conceived and designed the experiments: D-YG and J-ZL. Performed the experiments: D-YG and J-ZL. Analyzed the data: D-YG and XL. Wrote the manuscript: D-YG and J-ZL. Contributed materials and analysis tools: XL and Y-jL. Provided lab equipment for running the study: XL and Y-jL.

## Conflict of Interest Statement

The authors declare that the research was conducted in the absence of any commercial or financial relationships that could be construed as a potential conflict of interest.
